# Personalized cancer vaccination is emerging: lessons learnt from renal cancer and challenges for broader application

**DOI:** 10.1038/s41392-025-02204-z

**Published:** 2025-04-09

**Authors:** Elke Schaeffeler, Juliane Walz, Matthias Schwab

**Affiliations:** 1 Dr. Margarete Fischer-Bosch Institute of Clinical Pharmacology, Stuttgart, Germany; 2 Cluster of Excellence iFIT (EXC2180) “Image-Guided and Functionally Instructed Tumor Therapies”, University of Tuebingen, Tuebingen, Germany; 3 Department of Peptide-based Immunotherapy, Institute of Immunology, University and University Hospital Tübingen, Tübingen, Germany; 4 Departments of Clinical Pharmacology, Pharmacy and Biochemistry, University of Tuebingen, Tuebingen, Germany

**Keywords:** Oncology, Medical research

A recent phase I trial published in *Nature* by Braun et al.^1^ showed data on personalized cancer vaccination (PCV) in patients with high-risk, resected clear cell renal cell carcinoma (ccRCC) targeting neoantigens including to key RCC driver somatic mutations and resulting in antitumour immunity. Notably, recurrence was not observed in any of the nine patients at median follow-up of 40.2 months, as well as dose-limiting toxicities, which renders neoantigen-targeting PCV as promising RCC therapy in the adjuvant setting.

RCC consists of several histological subtypes like ccRCC, which is the most prevalent (>75%). Whereas 10% of patients already present with metastasis at date of diagnosis, about 10% will develop metastasis later. Partial or radical nephrectomy is used to treat early stage RCC resulting in cancer-specific survival rates of 95% after 5 years. Treatment of metastatic disease includes immune checkpoint inhibitors alone or in combination with tyrosine kinase inhibitors, which have improved survival rates in recent years. However, the long-term success rate in the metastatic setting remains unsatisfactory. The benefit of (neo)adjuvant immunotherapy to improve outcome in RCC is still limited and reliable biomarkers to identify patients at risk of worse outcome are lacking. Therefore, novel therapeutic strategies are needed, especially for patients at risk of recurrence.

**Fig. 1:**
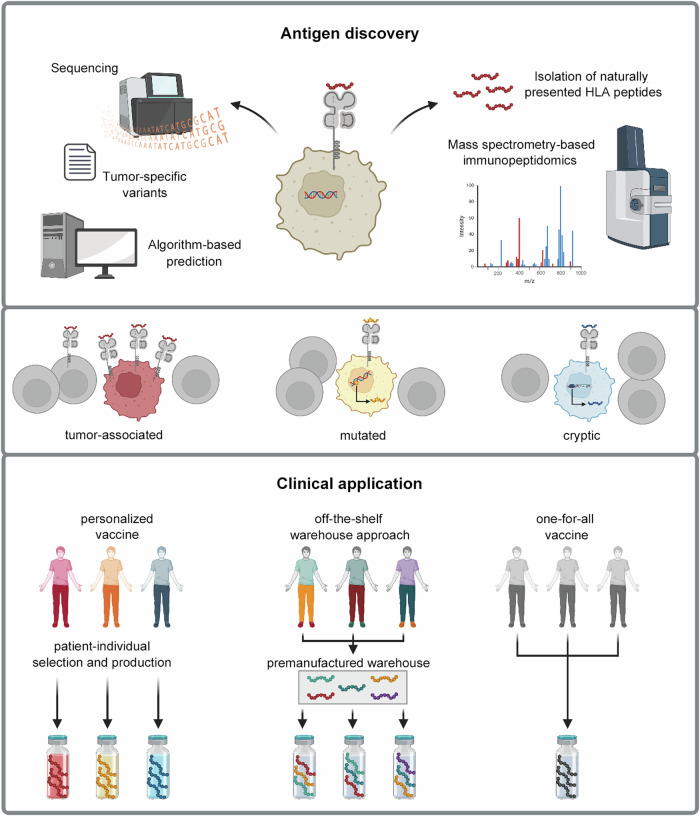
Cancer vaccination from antigen discovery to clinical application. Neoepitopes derived from cancer mutations used as antigen targets for cancer vaccination can either be predicted through next-generation genome/transcriptome sequencing approaches or a direct peptide target identification is feasible by mass spectrometry-based analysis, which allows the analysis of all naturally presented HLA ligands, termed the HLA ligandome or immunopeptidome of cancer cells. Promising targets for vaccination are classical neoepitopes derived from point- or frame-shift mutations, neoepitopes from gene fusions and non-mutated tumor-associated antigens with a high presentation frequency across multiple patients as well as the novel category of cryptic antigens. Different concepts for clinical vaccine application exist, namely personalized vaccines, warehouse-based personalization, or one-for-all vaccines. Figure created with BioRender.com

In this context, cancer vaccination offers great potential (Fig. 1). However, several challenges need to be overcome for widespread application, including the identification of suitable human leukocyte antigen (HLA)-presented tumor antigens. Current approaches primarily use cancer mutation-derived neoepitopes as antigen targets. However, intratumoral heterogeneity and the limited number of mutations presented as HLA-restricted neoepitopes on the tumor cell surface may complicate the identification of suitable targets through genome/transcriptome-based sequencing. Direct peptide target identification can be achieved by mass spectrometry-based analysis of all naturally presented HLA ligands, the so-called HLA ligandome/immunopeptidome of cancer cells.^2^ Beyond neoepitopes resulting from point or frame-shift mutations, this approach enables the identification of neoepitopes from gene fusions, non-mutated tumor-associated antigens with a high presentation frequency across multiple patients, arising from differential gene expression or protein processing in tumor cells, and the novel category of cryptic antigens arising from non-canonical gene products (Fig. 1).

Three different strategies with varying degrees of personalization have been proposed for the clinical application of cancer vaccines: personalized vaccines, warehouse-based personalization and off-the-shelf/one for all. (Fig. 1) Personalized vaccines are based on the on-demand de novo manufacturing of patient-specific products, individually tailored to the mutational profile and peptides presented on the patient’s tumor, allowing maximum benefit for each patient but with limitations for routine use. Patient-specific products, selected from pre-defined high-frequency tumor peptides, can be assembled using warehouse concepts. Depending on patient characteristics (e.g. mutation profile), the vaccine cocktail is individually assembled from separately manufactured peptides in a time- and cost-saving manner. Previously, off-the-shelf-based approaches provided vaccines for common HLA allotypes that were unsuitable for a significant proportion of patients. However, the characterization of high-frequency tumor antigens in a given tumor entity and the dissection of long HLA class II tumor peptides with promiscuous HLA binding with a variety of embedded HLA class I ligands, allowing their HLA allotype independent application, may reopen the door to one-for-all vaccine approaches.

Previous studies have aimed to identify promising targets for vaccination strategies in RCC. Reustle at al.^3^ identified HLA class I- and class II-presented peptides, that are tumor-specific and over-presented in ccRCC. Additionally, source genes were involved in tumor pathogenesis, supporting their potential as targets for ccRCC immunotherapy.^3^

So far, several vaccination studies in RCC have been conducted using autologous whole tumor cells, a single neoantigen-containing peptide, or tumor-associated antigen peptides. In contrast to previous studies, the study by Braun et al.^1^ evaluated neoantigens derived from tumor-specific mutations.

Generally, RCC represents one of the tumors with a low mutational burden. Main key drivers in ccRCC include the *VHL* tumor suppressor gene, *PBRM1*, *SETD2*, and *BAP1*. Braun et al.^1^ showed that effective vaccines in most patients contained peptides derived from at least one mutation in a driver gene. In addition, at least one peptide resulted from a frame-shift insertion or deletion subsequently leading to a novel open reading frame. The selected vaccine antigens proved to be immunogenic. Vaccination induced polyfunctional T cell responses (mainly CD4^+^T cell response), expansion of T cell clonotypes and immune reactivity against autologous tumor cells, but these data are limited by a very small sample size of only nine patients. In addition, other factors need to be taken into account when assessing the effectiveness of cancer vaccines, particularly in terms of long-term outcome and 5-year risk of disease recurrence, as only a short follow-up of a median of 40 months has been considered.

The vaccine composition and timing are also crucial. The adjuvant used by Braun et al.^1^ has been demonstrated to activate antigen presenting cells. Recently developed adjuvant formulations even enable a continuous immune stimulation and induction of anti-cancer T cell response persisting for several years upon single applications.^2,4^

Based on data from Braun et al.,^1^ PCVs seem to be encouraging in the adjuvant setting in the presence of minimal residual disease. This setting provides an optimal ratio of effector T cell to target cancer for the immune system to fight the tumor. PCVs may effectively clear residual tumor cells, resulting in long-term treatment response. In the case of advanced RCC, which is characterized by an immunosuppressive microenvironment, combination therapies may be considered. The study already applied the checkpoint inhibitor ipilimumab subcutaneously to a subset of patients in addition with PCVs, yielding only minor alterations in immediate immune response,^1^ which may be in part explained by the significantly lower ipilimumab dosage of 2.5 mg per injection compared to conventional systemic dosages. Interestingly, in contrast to findings of employing adjuvant pembrolizumab, no patient exhibited severe toxicity, regardless of additional administration of ipilimumab.

The comprehensive immunophenotyping of circulating proteins in plasma samples showed increased levels of angiogenesis markers and markers of T cell suppression after vaccination, providing first hypothesis for alternative drug combinations.^1^ Thus, to maximise the immune response, combining PCVs with targeted therapies (e.g. VEGF inhibitors) or even epigenetic modulators appears to be promising.

Future approaches may concentrate on reducing tumor load prior to vaccination or on overcoming factors such as tumor-mediated immunosuppression, immune escape mechanisms and impaired T cell infiltration to the tumor site. We showed that cancer drugs alter the immunopeptidome of tumor cells, resulting in the induction of cancer-specific drug-induced antigens as novel target structures, which may lead to novel avenues of synergistic vaccine-based cancer treatment.^5^ Further studies are needed to investigate alternative therapies to increase efficacy of cancer vaccines.
